# Construct of Carbon Nanotube-Supported Fe_2_O_3_ Hybrid Nanozyme by Atomic Layer Deposition for Highly Efficient Dopamine Sensing

**DOI:** 10.3389/fchem.2020.564968

**Published:** 2020-10-21

**Authors:** Yingchun Yang, Tao Li, Yong Qin, Lianbing Zhang, Yao Chen

**Affiliations:** School of Life Sciences, Northwestern Polytechnical University, Xi'an, China

**Keywords:** atomic layer deposition, hybrid nanozymes, ultrafine Fe_2_O_3_ nanoparticles, peroxidase activity, biosensing

## Abstract

The Fe_2_O_3_ nanozyme has been identified as the most promising alternative for the Fe_3_O_4_ nanozyme due to its relatively low toxic risk and good chemical stability. However, its enzyme-like activity is relatively low enough to meet specific application requirements. Furthermore, previous synthesis approaches have difficulties in fabricating ultra-small Fe_2_O_3_ nanoparticles with tunable size and suffer from agglomeration problems. In this study, atomic layer deposition (ALD) was used to deposit Fe_2_O_3_ on surfaces of carbon nanotubes to form hybrid nanozymes (Fe_2_O_3_/CNTs). ALD enables the preparation of ultrafine Fe_2_O_3_ nanoparticles with precise size control <1 nm, while CNTs could be served as promising support for good dispersibility and as an effective activity activator. Hence, the formed Fe_2_O_3_/CNTs exhibit excellent peroxidase-like activity with a specific peroxidase activity of 24.5 U mg^−1^. A colorimetric method for sensing dopamine (DA) was established and presented good sensitivity with a limit of detection (LOD) as low as 0.11 μM. These results demonstrated that, in virtue of meticulous engineering methods like ALD, carbon nanomaterial-based hybrids can be developed as talented enzyme mimetic, thus paving a way for nanozyme design with desired activity and broadening their applications in biosensing and other fields.

## Introduction

It is well-known that nanozymes have been developed as the most promising alternative for natural enzymes, owing to their good stability, simple and large-scale preparation, and cost effectiveness (Gao et al., 2007; Lin et al., 2014; Wang et al., 2019). In the past 10 years, over 300 kinds of nanomaterials, including metals, metal oxides, and carbon nanomaterials, have been demonstrated to mimic the activities of oxidase, peroxidase, catalase, and superoxide dismutase, which connects an important bridge between nanotechnology and biological science (Natalio et al., 2012; Wei and Wang, 2013; Liu and Liu, 2017; Li et al., 2018; Wu et al., 2019).

As the most studied nanozymes, iron oxide nanozymes (IONzymes) have attracted great interest since the first exciting discovery that ferromagnetic oxide possesses an intrinsic peroxidase activity (Gao et al., 2007), and they show great application potential in fields of biosensing, magnetic resonance imaging, anti-biofouling, and cancer therapy (Cheng et al., 2017; Jiang et al., 2019; Li et al., 2019; Wang et al., 2019; Šálek et al., 2020). Between the two main IONzymes Fe_3_O_4_ and Fe_2_O_3_, most attention was paid on Fe_3_O_4_ due to the relatively higher saturation magnetization and simpler synthesis procedures. However, the ferrous ions of Fe_3_O_4_ may raise its toxic risk and make it chemically unstable (Chen et al., 2012). Therefore, Fe_2_O_3_ nanozymes should be better candidates for applications. However, their enzyme-mimicking activities are relatively low enough to meet a variety of specific application requirements (e.g., biosensing, antimicrobial therapy). Furthermore, it remains a great challenge to synthesize ultrafine Fe_2_O_3_ nanoparticles with both controllable and uniform sizes, as well as to eliminate nanoparticle aggregation, which could inevitably affect their enzyme-like activity during catalytic reaction.

Among the huge family of nanozymes, great attention was also paid on carbon nanomaterials due to their excellent nanozymatic activities, diverse structures, and good biocompatibility (Sun et al., 2017; Wang et al., 2018). Especially, their structural merits such as large surface area and good mechanical properties make them an ideal support for anchoring metal/metal oxide nanozymes to form hybrid nanozymes and to inhibit the possible agglomeration of nanoparticles (Tao et al., 2013). Wang Q. et al. (2017) reported that Fe_3_O_4_ nanoparticles loaded on 3D porous graphene exhibited good dispersibility as well as stability. Moreover, their high electrical conductivity could facilitate electron transfer in many redox reactions, displaying a synergistic effect on the catalytic properties of metal/metal oxide (Yang et al., 2014). Such promotion effect was also applicable for upregulating enzyme-mimicking activities of metal/metal oxide nanozymes, thus leading to greatly enhanced activities compared to their single component. For instance, the peroxidase-like activity of Pt and Fe_3_O_4_ was remarkably increased when hybridized with carbon nanodots or C_3_N_4_ nanoflakes (Fan et al., 2018; Wang et al., 2018).

Recently, atomic layer deposition (ALD), as a gas-phase film deposition technology, has been demonstrated to be an advanced avenue in the preparation of ultrafine nanoparticles (Marichy and Pinna, 2013; Zhang and Qin, 2018). Owing to its self-limiting characteristic, it is capable of synthesizing nanoparticles with accurate size control at the atomic level. Zhang et al. reported that a series of Pt species, including single Pt atoms, Pt clusters, and Pt nanoparticles, could be easily fabricated by use of ALD (Sun et al., 2013). Furthermore, ALD can be served as a powerful approach to prepare hybrid nanomaterials due to its good step coverage over substrates with a complicated structure (Marichy and Pinna, 2013). Our previous study found that carbon nanotubes and graphene-supported nanoparticles with adjustable size and good distribution can be achieved by ALD (Zhang et al., 2015, 2016, 2018), which cannot be fulfilled by traditional method and confirms again the superiority of ALD. Furthermore, it has been demonstrated that the interface structure of nanozymes could also be precisely engineered by ALD to tune their enzyme-mimicking activities (Chen et al., 2020). However, until now there is no report on fabricating carbon-based IONzymes by ALD.

Hence, in this study, CNT-supported Fe_2_O_3_ nanozymes (Fe_2_O_3_/CNTs) were fabricated to solve the problems of aggregation and relatively low activity of the Fe_2_O_3_ nanozyme. ALD was adopted, for the first time, to precisely synthesize CNT-supported Fe_2_O_3_ nanoparticles with good uniformity and dispersibility. With the adjustment of cycle numbers, ultrasmall Fe_2_O_3_ nanoparticles with size down to 1 nm could be accurately achieved. The uniform and ultrafine Fe_2_O_3_ nanoparticles, as well as the synergistic effect of CNTs, contributed to an excellent enzyme-mimicking activity of the Fe_2_O_3_/CNT hybrid nanozyme, which made it an efficient peroxidase mimic for the catalytic conversion of a chromogenic substrate of 3,3′,5,5′-tetramethylbenzidine (TMB) and for colorimetric probe in dopamine (DA) sensing.

## Materials and Methods

### Materials

Carbon nanotubes (CNTs) with diameter of 20–30 nm and length of 0.5–2 μm were purchased from Shenzhen Nanotech Port Co., Ltd. (China). Raw CNTs were treated in HNO_3_ (68 wt %) at 140°C for 4 h to remove possible catalyst residues and provide nuclear sites for subsequent ALD processes. Ferrocene, dimethyl sulfoxide (DMSO), hydrogen peroxide (H_2_O_2_), dopamine, and 3,3′,5,5′-tetramethylbenzidine (TMB) were obtained from J&K Scientific. All of the chemicals were used as received.

### Synthesis of Fe_2_O_3_/CNTs by ALD

Firstly, treated CNTs were dispersed in ethanol under ultrasonic agitation and dropped onto quartz wafers. Then, the wafers were placed into an ALD chamber and the ALD process was carried out in a homemade and self-heating system. For Fe_2_O_3_ ALD, both ferrocene and O_3_ were used as precursors and ferrocene was heated to 75°C to obtain optimal vapor pressure. To ensure successful Fe_2_O_3_ deposition, the chamber was maintained at 260°C. The pulse, exposure, and purge time for ferrocene and O_3_ were 1.5/20/30 and 0.2/15/30 (s), respectively. The particle size of Fe_2_O_3_ was adjusted by ALD cycles and the samples with different ALD cycle numbers (n) were noted as nFe_2_O_3_/CNTs in this study. After deposition, the samples were collected for further use.

### Characterization

Transmission electron microscopy (TEM) and high-resolution TEM (HRTEM) were measured on a FEI Tecnai F20 instrument. High-angle annular dark-field scanning-TEM (HAADF-STEM) images were also collected on this equipment operated at 200 kV. X-ray photoelectron spectroscopy (XPS) data were collected with a Thermo ESCALAB 250 xi with an Al-Ka line as the radiation source. X-ray diffraction (XRD) data were recorded on a MAXima XRD-7000 diffractometer with Cu Kα radiation. Inductively coupled plasma optical emission spectrometry (ICP-OES; Thermo iCAP 6300) was used to determine the content of the metal in the hybrid nanozyme.

### Peroxidase-Mimicking Activity Assay

A colorimetric method was adopted to estimate the peroxidase activity of Fe_2_O_3_/CNT samples by using TMB as the chromogenic substrate. Typically, the catalytic reaction was processed by adding 20 μL of Fe_2_O_3_/CNT nanozyme (0.5 mg/mL), 80 μL of TMB (2.5 mM), and 100 μL of H_2_O_2_ (10 mM) into a 2 mL tube. The final volume was adjusted to 1 mL by adding 800 μL NaAc buffer (0.2 M, pH 3.6). The mixed solutions were incubated at 37°C for 10 min. Then, the UV-vis absorbance of oxidized TMB (TMB_ox_) at a wavelength of 652 nm was immediately recorded. Furthermore, the enzyme-like property was evaluated according to Yan's protocol (Jiang et al., 2018). The experiments were carried out at 37°C in NaAc-HAc buffer (0.2 M, pH 4.0) containing TMB (4 μL of 10 mg mL^−1^) and H_2_O_2_ (1 M). The time-dependent absorbance curves were immediately recorded at a 10 s interval within 400 s, and the nanozyme activity expressed in units (U) was calculated according to the following equation:

(1) $$\begin{array}{l} b_{\text{nanozyme}}=\;\frac{V}{\varepsilon l}\times \frac{\Delta A}{\Delta t} \end{array}$$

where *b*_nanozyme_ is the nanozyme activity (U), *V* is the total volume of reaction solution (μL), ε is the molar absorption coefficient of the TMB substrate (39,000 M^−1^ cm^−1^ at 652 nm), *l* is the optical path length through reaction solution (cm), and Δ*A*/Δ*t* is the initial rate of the absorbance change (per minute). The specific activity of the nanozyme was determined using the following equation:

(2) $$\begin{array}{l} \;a_{\text{nanozyme}}=\;\frac{b_{\text{nanozyme}}}{m} \end{array}$$

where *a*_nanozyme_ is the specific activity of the nanozyme (U mg^−1^) and *m* is the nanozyme amount for each assay (mg).

### Steady-State Kinetic Analysis

Kinetic experiments were carried out in a 2 mL cuvette with 1 mL NaAc buffer (0.2 M, pH 3.6) containing 10 μg 10Fe_2_O_3_/CNTs. Both TMB and H_2_O_2_ were also added and tested in a time-course model at 37°C. TMB_ox_ at 652 nm was recorded every 30 s in the range of 0–10 min, and the initial rates of two different substrates were determined. The kinetic assay with TMB as substrate was conducted by varying concentrations of TMB with the concentration of H_2_O_2_ fixed at 0.1 nM, and vice versa. The key kinetic parameters, such as Michaelis–Menten constant (*K*_*m*_), catalytic efficiency (*K*_*cat*_), and specific activity were calculated by fitting the initial velocity data to the Michaelis–Menten equation: *1/V* = *K*_*m*_*/V*_*m*_ (*1/[S]*+ *1/K*_*m*_), where *V* represents the initial rate, *V*_*m*_ is the maximal rate of the enzyme-like reaction, *K*_*m*_ corresponds to the Michaelis–Menten constant, and [*S*] is the substrate concentration.

### Colorimetric Sensing of DA

Colorimetric sensing of DA was evaluated using the 10Fe_2_O_3_/CNTs hybrid nanozyme under the optimized conditions. The experiments were carried out as follows: 80 μL of TMB (0.2 mM in DMSO), 100 μL of H_2_O_2_ (0.1 mM), and 5 μL of 10Fe_2_O_3_/CNTs (0.5 mg/mL) were successively added to 750 μL of NaAc buffer (0.2 M, pH 3.6). Then, 50 μL of DA with different concentration was added and the mixture was incubated at 37°C for 10 min, followed by monitoring of the UV-vis absorbance of TMB_ox_ at 652 nm.

## Results and Discussion

**Figure 1:**
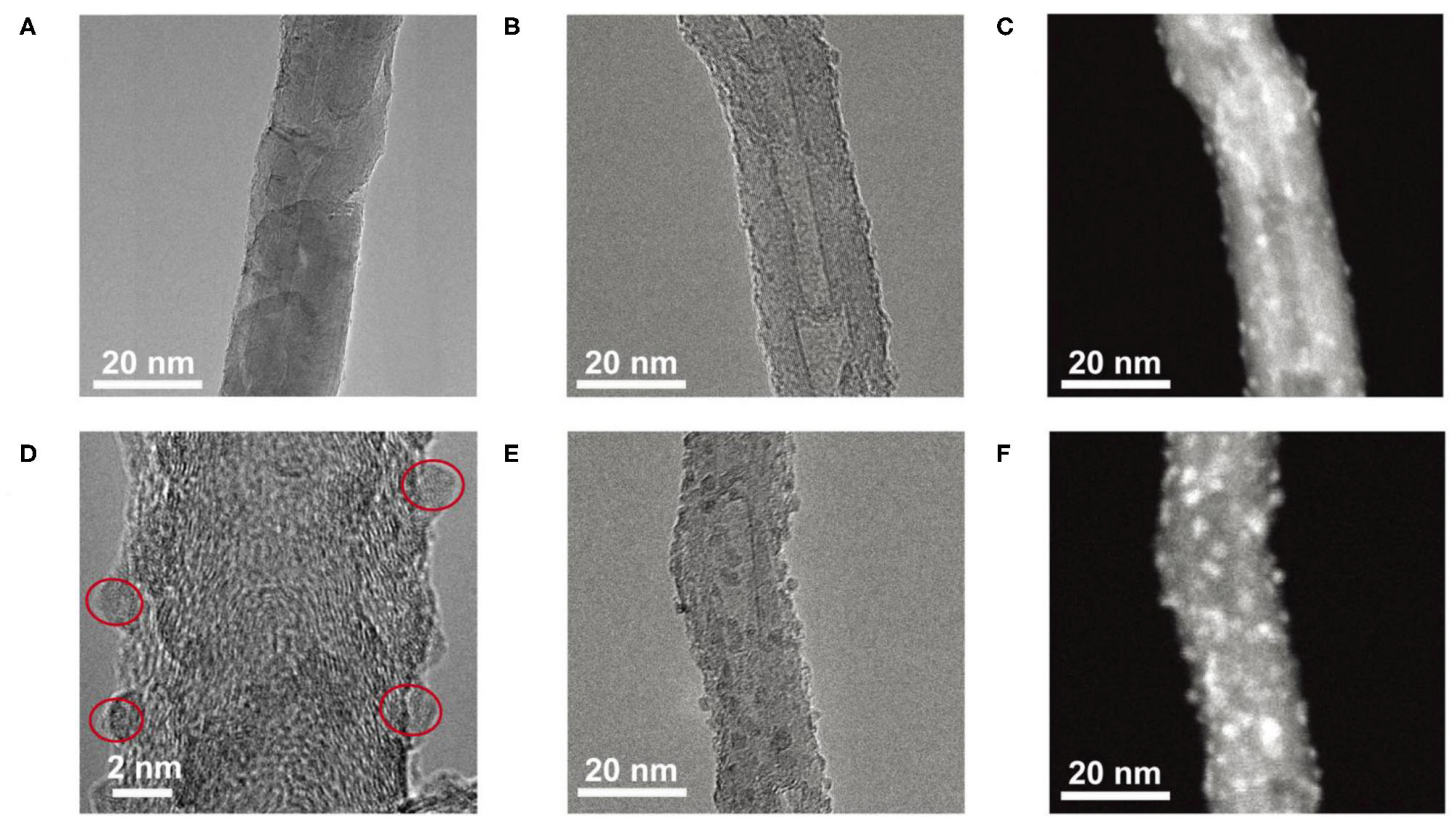
TEM images of 5Fe_2_O_3_/CNTs **(A)**, TEM image **(B)**, HAADF image **(C)**, HRTEM image of 10Fe_2_O_3_/CNTs **(D)**, HRTEM image **(E)**, and HAADF image **(F)** of 15Fe_2_O_3_/CNTs.

In order to characterize the morphology of the as-deposited Fe_2_O_3_/CNT samples, TEM measurements were conducted; the results are depicted in Figure 1. For the sample of 5Fe_2_O_3_/CNTs, almost no Fe_2_O_3_ nanoparticle can be distinguished from surfaces of CNTs due to its low loading content (Figure 1A). With increasing ALD cycles, it remained difficult to identify the existence of Fe_2_O_3_ in the TEM image of 10Fe_2_O_3_/CNTs (Figure 1B), but the HAADF image (Figure 1C) recorded at the same region reveals visibly the successful deposition of Fe_2_O_3_ nanoparticles on CNTs. This can be clearly distinguished from the HRTEM image as depicted in Figure 1D. It is obvious that these nanoparticles are uniformly distributed with diameter of around 1 nm. In addition, it is worth noting that the as-synthesized Fe_2_O_3_ by ALD is amorphous with no visible crystalline structure found in HRTEM images. When 15 cycles of Fe_2_O_3_ were applied, a higher density of nanoparticles with larger size of around 2 nm on CNTs was observed both in HRTEM (Figure 1E) and HAADF (Figure 1F) images. These results fully demonstrate that ALD can be served as a novel avenue to synthesize Fe_2_O_3_ nanoparticles with ultrasmall and adjustable size, which cannot be fulfilled by traditional method and fully reveals the superiority of ALD.

**Figure 2:**
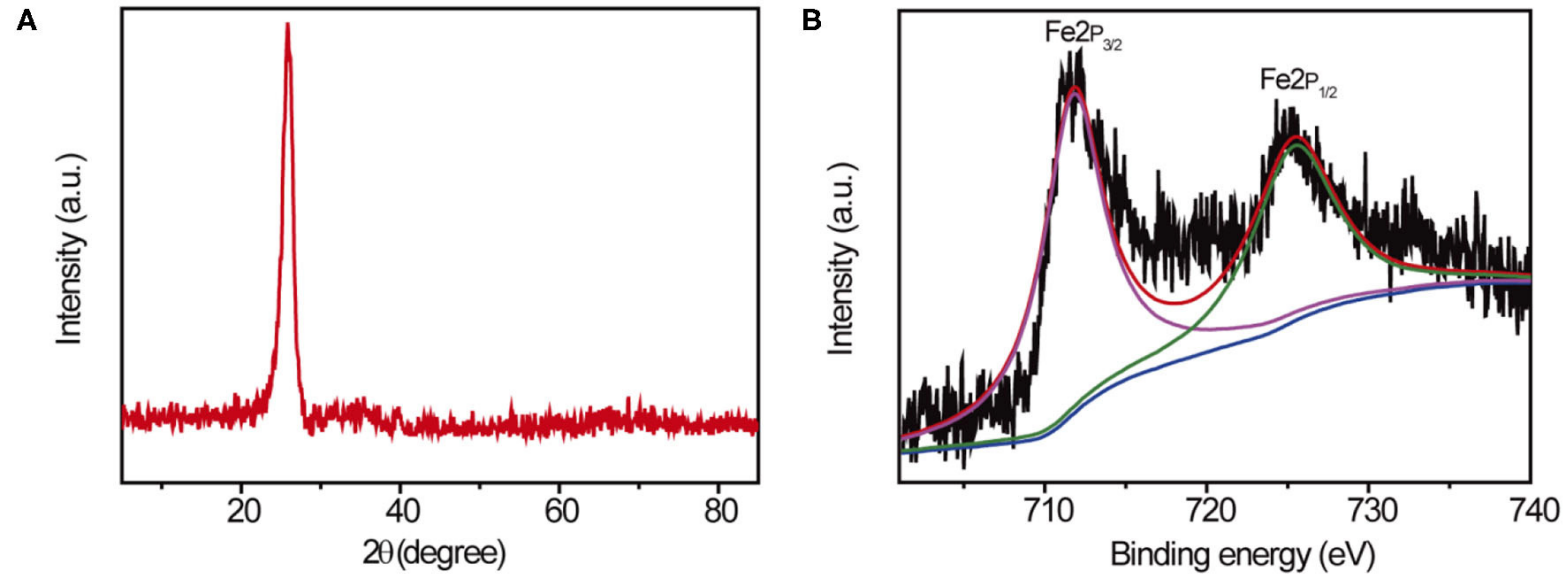
**(A)** XRD spectra of Fe_2_O_3_/CNTs and **(B)** the XPS core level of Fe2p.

Further studies were conducted in characterization of the chemical structure of 10Fe_2_O_3_/CNTs by XRD measurement, and the spectra of 10Fe_2_O_3_/CNTs are presented in Figure 2A. The main diffraction peak located at 2θ = 23.8° belongs to typical XRD spectra of CNTs. However, no characteristic peak of iron oxide could be found in the spectra, which matches well with the abovementioned TEM results. As revealed by ICP analysis, the content of Fe_2_O_3_ in 10Fe_2_O_3_/CNTs was as low as 2.1 wt% (results not provided). Actually, similar results have been achieved in our previous study (Zhang et al., 2015) and the unobservable XRD signal of Fe_2_O_3_ was ascribed to its poor crystallinity and low content, when few cycles of ALD were conducted. In addition, XPS test was also performed to investigate the surface elemental composition of 10Fe_2_O_3_/CNTs. Deconvolution of Fe2p core-level spectra shown in Figure 2B reveals that there are two main peaks located at 712.6 and 726.1 eV, which can be ascribed to Fe2p 3/2 and Fe2p 1/2, respectively. These results demonstrated the 3+ valence state of Fe species in 10Fe_2_O_3_/CNTs. Meanwhile, the existence of an associate satellite peak located at 718.9 eV could correspond to the characteristic XPS spectra of Fe_2_O_3_. The combined results of TEM, XRD, and XPS strongly support that Fe_2_O_3_ was successfully deposited on CNTs by ALD.

### The Peroxidase-Mimicking Activity of Fe_2_O_3_/CNTs

**Figure 3:**
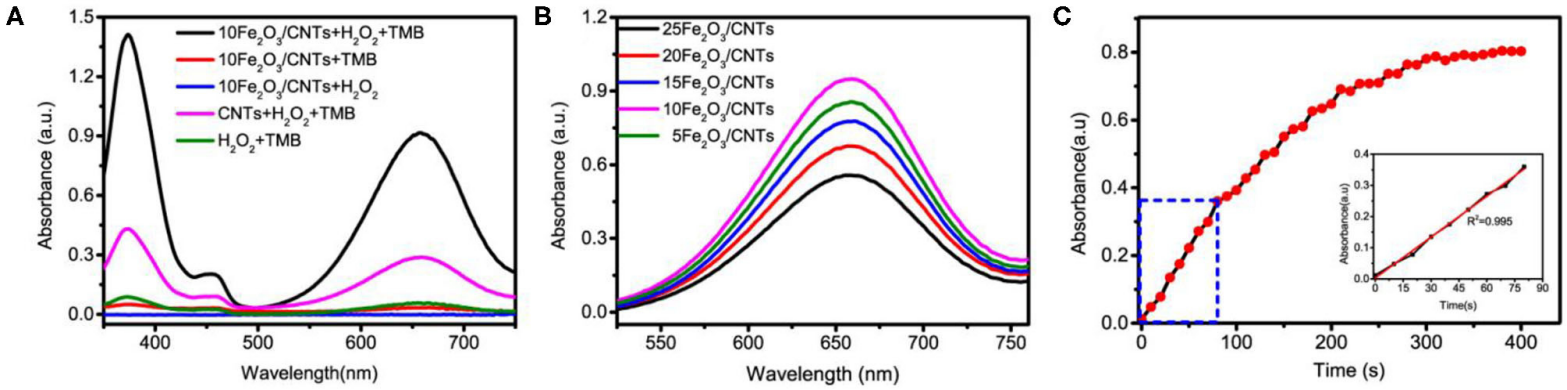
**(A)** UV-vis spectra of monitoring oxidized TMB catalyzed by different systems. **(B)** Peroxidase-like activities of Fe_2_O_3_/CNTs with different Fe_2_O_3_ cycles. **(C)** The absorbance-time curves of the TMB chromogenic reaction catalyzed by the 10Fe_2_O_3_/CNTs.

With assistance of the ALD method, it is expected that the as-synthesized Fe_2_O_3_/CNT samples with ultrafine particle size and good dispersibility possess excellent enzyme-mimicking activities. The peroxidase-like activities of Fe_2_O_3_/CNT samples were manifested through TMB oxidation in the presence of H_2_O_2_ into a charge transfer product (TMB_ox_). Several control experiments were carried out to confirm the catalytic nature. It can be seen in Figure 3A that neither H_2_O_2_ nor TMB alone was capable of inducing catalytic reaction and no significant absorbance peak was generated. When both substrates were presented, the catalytic oxidation rate was accelerated by 10Fe_2_O_3_/CNTs in comparison to bare CNTs, as revealed by a great enhancement in the absorbance at 652 nm. It can be concluded that the hybridization of CNTs with uniform and ultra-small Fe_2_O_3_ nanoparticles contributes to great enhancement in peroxidase-like activity.

Since ALD has a unique advantage of flexible control over cycles, it enables the optimization of the peroxidase-mimicking activity by adjusting the particle size of Fe_2_O_3_. The peroxidase-like activity of Fe_2_O_3_/CNT samples with different cycles was also tested, and the results are shown in Figure 3B. It is obvious that the activity was enhanced with increase in ALD cycle (*n* < 10) but showed an opposite trend when more than 10 cycles of Fe_2_O_3_ ALD were conducted. Among them, 10Fe_2_O_3_/CNTs presented the highest activity, which might be due to the hybridization of CNTs with Fe_2_O_3_, as well as the ultrafine nanoparticles with good distribution. The relative low activity of 5Fe_2_O_3_/CNTs might be attributed to the low loading content of Fe_2_O_3_ in the hybrid nanozyme, while the nanoparticle stacking as well as the blocking access to active sites resulted in the low activity of samples with more than 10 cycles. As depicted in Figure 3C, the specific activity determined according to the protocol of Yan's group (Jiang et al., 2018) shows that the absorbance is linear to reaction time in the first minutes. By choosing 80 s as the initial rate period, the calculated peroxidase activity of 10Fe_2_O_3_/CNTs was 24.5 U mg^−1^ (the inset in Figure 3C). It is obvious that this value is higher than that of reported IONzymes and carbon-based nanozymes (Table 1) and very close to the highest specific activity of the Fe–N–C single atom nanozyme, demonstrating the excellent performance of 10Fe_2_O_3_/CNTs fabricated by ALD.

**Table 1:** Typical specific peroxidase-like activity of 10Fe_2_O_3_/CNTs and its comparison with other nanozymes.

**Nanozymes**	**Specific peroxidase activity (U mg^**−1**^)**	**References**
Fe_3_O_4_ NPs	5.143	Jiang et al., 2018
IONPs	8.5	Šálek et al., 2020
Carbon NPs	3.302	Jiang et al., 2018
Fe–N–C	57.76	Niu et al., 2019
Fe–N–C	25.33	Jiao et al., 2020
This work	24.5	–

### Steady-State Kinetic Theory of 10Fe_2_O_3_/CNTs

**Figure 4:**
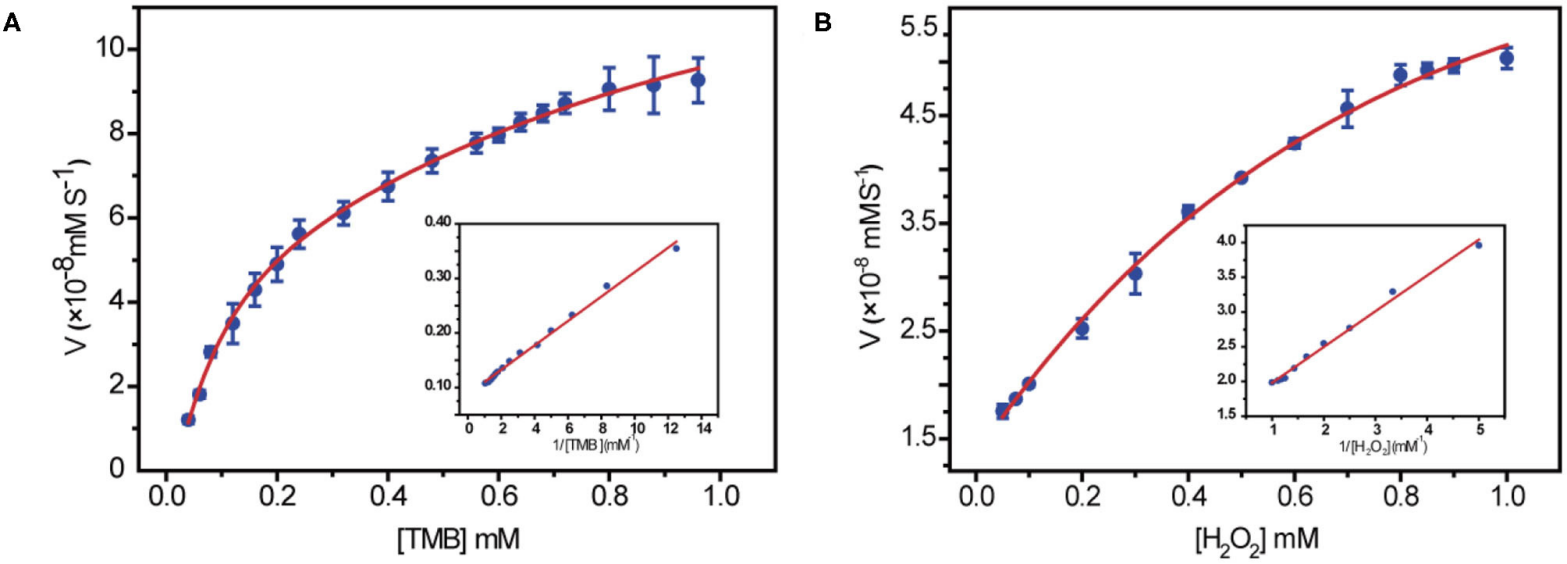
Steady-state kinetic assays by using 10 μg/mL 10Fe_2_O_3_/CNTs in 0.2 M NaAc (pH 3.6) at 37°C for 10 min. **(A)** The concentration of H_2_O_2_ was 0.5 mM, and the TMB concentration was varied. **(B)** The concentration of TMB was 0.4 mM, and the H_2_O_2_ concentration was varied. Insets are the Lineweaver–Burk plots of the double reciprocal of the Michaelis–Menten equation, with the concentration of one substrate fixed and the other varied. Error bars shown represent the standard error derived from three repeated measurements.

In order to get a clear understanding of the intrinsic mechanism for peroxidase activity enhancement of 10Fe_2_O_3_/CNT samples, the kinetic was exploited by altering the concentration of one substrate while keeping constant of the other. A set of concentrations for each substrate was recorded in a time-course mode, and the initial rate of TMB and H_2_O_2_, which is the slope of ΔA652 per unit time (min), was obtained by applying the Beer–Lambert law. The collected data were fitted based on the Michaelis–Menten equation, and the nanozymatic parameters were calculated with the typical Lineweaver–Burk double reciprocal plots as depicted in Figure 4.

The key parameters of *K*_*m*_ and *V*_*max*_ are listed in Table 2. Since *K*_*m*_ is identified as the indicator of the affinity of enzyme to substrates, achieving low *K*_*m*_ and high *V*_*max*_ is important to ensure better catalytic performance. It is obvious that the 10Fe_2_O_3_/CNTs displayed smaller *K*_*m*_ values for H_2_O_2_, indicating a better affinity to H_2_O_2_ and a relatively low concentration of H_2_O_2_ needed for achieving a high response of 10Fe_2_O_3_/CNTs in catalytic reaction. This result is consistent with the report that in the reaction of TMB oxidation with H_2_O_2_, decomposition of H_2_O_2_ is the rate-determining step (Zhao et al., 2008), whereby the *K*_*m*_ for TMB is close to the natural HRP enzyme, suggesting its good binding affinity for TMB.

**Table 2:** Typical Michaelis–Menten constant and maximum velocity for H_2_O_2_ and TMB substrates and their comparison with HRP.

**Catalyst**	**Substrate**	***K_***m***_* [mM]**	***V_***max***_* [10^**−8**^ Ms^**−1**^]**
10Fe_2_O_3_/CNTs	TMB	0.515	14.61
10Fe_2_O_3_/CNTs	H_2_O_2_	0.704	17.43
HRP (Gao et al., 2007)	TMB	0.43	10
HRP (Gao et al., 2007)	H_2_O_2_	3.7	8.71

### Sensitivity of 10Fe_2_O_3_/CNTs for Detection of DA

**Figure 5:**
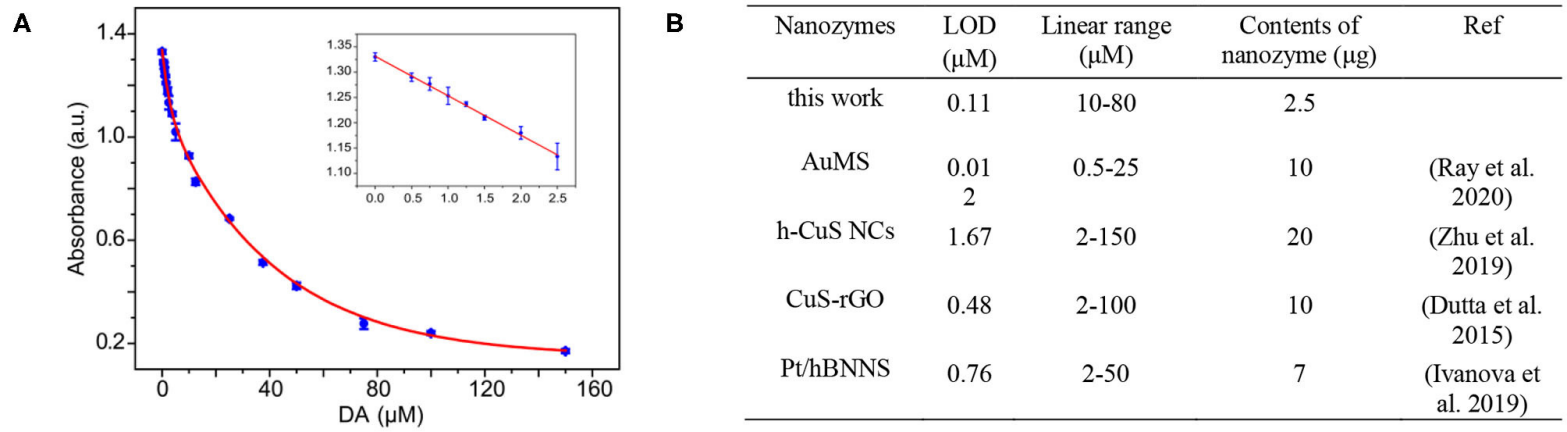
**(A)** The absorbance of oxidized TMB in the DA detection system in the presence of DA. (Insets) The linear calibration plot and the optical image for DA detection using 10Fe_2_O_3_/CNTs. The error bars indicate the standard deviation of three repeated measurements. **(B)** Comparison of the sensing properties of typical nanozymes (Dutta et al., 2015; Ivanova et al., 2019; Zhu et al., 2019; Ray et al., 2020).

A variety of biosensors have been established based on the excellent enzyme-mimicking activities of nanozymes. As an essential neurotransmitter, dopamine (DA) is critically involved in a variety of motor and non-motor information transmission and affecting human emotions and perceptions (Paval, 2017; Sgambato-Faure and Tremblay, 2018). DA disorder will cause a series of diseases (Ashok et al., 2017; Sgambato-Faure and Tremblay, 2018). For instance, if too much dopamine is secreted, it can lead to neurological dysfunction. Hence, it is necessary to develop a simple and sensitive approach to detect the DA level. Inspired by the behavior that the existence DA in catalytic reaction solution will inhibit peroxidase-mimicking activity of the 10Fe_2_O_3_/CNTs, a colorimetric method was employed to determination of DA. Figure 5A shows the absorbance response of TMB_ox_ in the system when different concentrations of DA were added. A typical linear calibration plot was obtained in the 0–25 μM concentration range with a limit of detection (LOD) of 0.11 μM. The LOD was calculated by using the typical formula LOD = 3σ/*k*, where σ is the standard deviation for the target-blank sample and *k* stands for the slope of the calibration curve. This approach provides a convenient and sensitive method for sensing of DA. As shown in Figure 5B, the LOD of our system is not the lowest by comparing with typical Au nanoparticles, hollow CuS nanocubes, and Pt nanoparticles. However, it is worth noting that the concentration of the nanozyme used in our approach is the lowest, which demonstrates that the 10Fe_2_O_3_/CNT hybrids fabricated by ALD are a promising candidate for DA biosensing applications.

## Conclusions

To sum up, CNT-loaded Fe_2_O_3_ nanoparticles with uniform distribution and precise size control can be easily prepared by ALD. The CNTs not only serve as the support to inhibit possible aggregation of Fe_2_O_3_ nanoparticles but also act as activity enhancer for Fe_2_O_3_, which endows Fe_2_O_3_/CNT hybrids with an excellent peroxidase activity. In our approach, the peroxidase activity could be optimized by adjusting the cycle number of Fe_2_O_3_ ALD and the highest activity was achieved by 10Fe_2_O_3_/CNTs, due to the well-distributed and ultrafine Fe_2_O_3_ nanoparticles on the surface of CNTs. The steady kinetic assay demonstrated that 10Fe_2_O_3_/CNTs show good binding affinity to both TMB and H_2_O_2_. In addition, a colorimetric method for sensing of DA was established based on the excellent activity of 10Fe_2_O_3_/CNTs, which presented a good sensitivity with LOD as low as 0.11 μM. Based on the abovementioned results, the unique advantage of ALD by precise and controllable nanomaterial fabrication enables a novel avenue for nanozyme synthesis with finely tunable activities, which can be convenient for in-depth investigation and understanding of the catalytic mechanism of nanozymes and broaden also their application in biosensing and other areas.

## Data Availability Statement

All datasets presented in this study are included in the article/supplementary material.

## Author Contributions

YY, TL, LZ, YQ, and YC conceived and carried out experiments, analyzed data, and wrote the paper. All authors read and approved the final manuscript.

## Conflict of Interest

The authors declare that the research was conducted in the absence of any commercial or financial relationships that could be construed as a potential conflict of interest.
